# Non-invasive phenotyping and drug testing in single cardiomyocytes or beta-cells by calcium imaging and optogenetics

**DOI:** 10.1371/journal.pone.0174181

**Published:** 2017-04-05

**Authors:** Yu-Fen Chang, Connor N. Broyles, Frances A. Brook, Mark J. Davies, Cameron W. Turtle, Takeharu Nagai, Matthew J. Daniels

**Affiliations:** 1 Division of Cardiovascular Medicine, Radcliffe Department of Medicine, University of Oxford, Oxford, United Kingdom; 2 Department of Biotechnology, Graduate School of Engineering, Osaka University, Suita, Osaka, Japan; 3 Department of Biotechnology, School of Engineering, Osaka University, Suita, Osaka, Japan; 4 Department of Biomolecular Science and Engineering, The Institute of Scientific and Industrial Research, Osaka University, Ibaraki, Osaka, Japan; 5 Department of Cardiology, Oxford University NHS Hospitals Trust, Oxford, United Kingdom; 6 BHF Centre of Regenerative Medicine, University of Oxford, Oxford, United Kingdom; University of Kansas Medical Center, UNITED STATES

## Abstract

Identification of drug induced electrical instability of the heart curtails development, and introduction, of potentially proarrhythmic drugs. This problem usually requires complimentary contact based approaches such as patch-clamp electrophysiology combined with field stimulation electrodes to observe and control the cell. This produces data with high signal to noise but requires direct physical contact generally preventing high-throughput, or prolonged, phenotyping of single cells or tissues. Combining genetically encoded optogenetic control and spectrally compatible calcium indicator tools into a single adenoviral vector allows the analogous capability for cell control with simultaneous cellular phenotyping without the need for contact. This combination can be applied to single rodent primary adult cardiomyocytes, and human stem cell derived cardiomyocytes, enabling contactless small molecule evaluation for inhibitors of sodium, potassium and calcium channels suggesting it may be useful for early toxicity work. In pancreatic beta-cells it reveals the effects of glucose and the K_ATP_ inhibitor gliclazide.

## Introduction

Disrupting the ion channels responsible for depolarisation and repolarisation of cardiomyocytes by gene mutation or off-target drug effect increases the risk of sudden arrhythmic death [1]. This pillar of safety pharmacology is principally studied using animal explant material and manual approaches requiring direct contact with the cell. Improvements to increase human relevance and experimental throughput are currently being evaluated in the Comprehensive In Vitro Proarrhythmia Assay (CiPA) initiative [2]. This multi-disciplinary industry, academia, and regulatory collaboration is testing strategies (e.g. automated patching platforms, in silico modelling), and substrates (e.g. human stem cell derived cardiomyocytes (hSC-CM) with the higher through-put methods that have been used to study repolarization changes (e.g. Multi-electrode array, or optical recordings of voltage or calcium) in response to known drugs or mutations [3].

The time taken for ventricular cells to depolarise and repolarise can be measured at the bedside by the QT interval on an electrocardiogram. Abnormal QT duration reveals inherited (Long/Short QT) or acquired disease states. Since medications were shown to cause QT prolongation, and sudden death [4], evolution of safety pharmacology and statutory medicines regulatory frameworks [5] limit such entities reaching the clinic. Detection of QT prolongation pre-clinically requires measurements reflecting the action potential duration (APD). Since the APD is influenced by beat frequency [6] methods to control the cell are also needed. Normally this is achieved with electrical stimulation and patch clamp. These require contact with the cell and with the dish, constraining throughput. Automated platforms offer modest improvement generating 150–3000 data points a day [7]. Adopting approaches to measure, and control, cells with light may eliminate the complexity required of patching, or the variability and toxicity of electrical field stimulation [8] and thus liberate the need for contact which constrains scalability.

Optogenetic tools, in place of electrical stimulation, can impose control on whole hearts [9,10], monolayer cultures of neonatal rodent cardiomyocytes [11–13] or hSC-CM’s [14,15]. Since genetically encoded indicators can be used to phenotype voltage or calcium transients in hSC-CM’s with less toxicity than chemical dyes [16,17] we considered combining approaches into an all-optical-all-genetic single cell assay as previously reported for cultured single neurons using compatible control/indicator pairs for voltage [18] or calcium [19–21]. In contrast to genetically encoded calcium indicators, equivalent voltage tools have a smaller dynamic range and signal to noise ratio, require cofactor (eg retinal) addition which may have biological consequences in SC-CM’s [13,22], and high power illumination which may limit observation duration. Whilst the calcium transient duration (CTD) is only a surrogate for the voltage changes that defines the APD; this integrated response from multiple currents and pumps closely reflects the cellular APD [23]. Hence we asked if optical control with calcium imaging may be possible, and useful, in single cells directly avoiding the need for monolayers to make single cell measurements.

## Materials and methods

### Construct and virus production

ChETA_TC_-5xMyc linked by a 2A peptide to R-GECO1 was codon optimised for human cells and synthesised (Genscript, Picastaway, NJ, US) and subcloned into the pDUAL backbone for commercial Adenoviral production (Vector Biolabs, Malvern, PA, US). Sequences are provided in S1 Appendix.

### Primary cardiomyocyte isolation

This investigation was approved by the Animal Welfare and Ethical Review Board at the University of Oxford and conforms to the UK Animals (Scientific Procedures) Act, 1986, incorporating Directive 2010/63/EU of the European Parliament. Adult guinea pigs were obtained from Harlan UK and sacrificed by cervical dislocation. Guinea pig left ventricular myocytes were isolated using an enzymatic digestion technique. Animals were culled via cervical dislocation and the heart was submitted to retrograde perfusion with a Ca^2+^ free buffer for 3 minutes followed by a 1mg/ml Collagenase type II solution (250 units/mg, Worthington Biochemical Corporation) for 9 minutes. The digested tissue was subsequently mechanically agitated in enzymatic solution for an additional 5 minutes and the cells were collected by centrifugation at low speed (500 rpm).

### ChETA_TC_-myc and R-GECO imaging in fixed cells

Adult ventricular cardiomyocytes were infected at an MOI of 5 as for live imaging and kept in storage solution in a humidified incubator at 37°C, 5% CO2. At 48 hours an aliquot was taken and paraformaldehyde to a final concentration of 4% was added, at 10 minutes, cells were pelleted (200rpm, 2min) in a benchtop centrifuge, washed with 1xPBS and resuspended in PBS. Cells were spun (Cytospin, Thermo Scientific) onto glass slides, and ringed with a PAP pen (Sigma). Permeabilisation with 0.1% Triton-X-100 in Tris Buffered saline for 10 minutes at room temperature was followed by blocking (0.2% albumin in permeabilisation buffer) for 20 minutes. Primary antibodies (mouse monoclonal 9E10 anti-myc (Santa-Cruz), and rabbit polyclonal anti-DsRed (Clontech) were diluted 1:200 in blocking buffer. Three hours after primary incubation cells were washed in permeabilisation buffer, and counter stained with Alexa-488 anti-mouse, and Alexa-568 anti-rabbit fab fragment secondaries (Invitrogen), nuclear counterstaining was with Topro3 (Invitrogen), for an hour before washing and mounting (Vectashield, Vector labs). Images were acquired on a Leica SP5 confocal microscope with a 63x oil immersion lens. hSC-CM’s were plated onto 0 thickness coverglass, infected at an MOI of 5. Cells were fixed and stained 48 hours after infection as above.

### Live cell imaging microscope

An inverted IX81 frame (Olympus, Japan), with a custom 7 LED array (Cairn Research, Faversham, UK), automated stage (Prior Scientific, Cambridge, UK), lens turret, and filter wheel is housed in a custom heated, humidified chamber (Solent Scientific, Segensworth, UK) with image collection on two C-1900 EMCCD cameras (Hamamatsu, Japan) mounted on a beam splitter (Photometrics, UK). Single camera mode image acquisition through an Olympus PlanApo N 60x oil objective lens (NA 1.42) using CellR software (Olympus) was used. A 30mW 405nm blue diode laser FRAP module (Olympus) was used at 10% power for cell stimulation, controlled through CellR. Software conflicts allowed a maximum programmed stimulation rate within CellR of 2.4Hz (144 beats per minute), hence factors of 2.4Hz were used in the stimulation series.

### Cardiomyocyte optical control and phenotyping

Primary cardiomyocytes were seeded onto coverslips and mounted in a Ludin chamber (Life Imaging Services GmbH, Switzerland) with Tyrode’s buffer. Electrical stimulation was achieved with a custom modification to the RC-37WS electrode (Warner Instruments) and myopacer unit (Ionoptix). Optical stimulation was achieved with the 405nm laser light at a power density of 1.17mW/mm^2^. Uninterrupted recording of the calcium transient was achieved with a 5msec 568nm LED illumination pulse. The RFP filter set (DS/FF01-560/25-25, T565lpxr dichroic mirror, and ET620/60 emission filter) was used to observe R-GECO.

### Stem cell derived cardiomyocyte optical control and calcium imaging

Human iPS derived cardiomyocytes and maintenance media were purchased from Axol Bioscience, (Cambridge, UK), and handled according to manufacturer’s recommendations at 37°C, 5% CO2 in a humidified incubator. After a week in culture they were singularised and re-plated onto Fibronectin (0.5%)/Gelatin (0.1%) coated 24-well MatTek plates (Ashland, MA, US) at 10,000 cells/well. Viral transduction at an MOI of 5 for 24 hours preceded imaging by 2 days. Results shown are obtained from a minimum of three separate batches of cells.

### Stem cell derived cardiomyocyte and chemical calcium dye imaging

iPS derived cardiomyocytes were plated out as above, and then loaded with 5μM Fluo-4-AM (Thermo-Fisher, UK) at room temperature for 20 min, free dye was washed off by media replacement with pre-heated culture media, followed by imaging with C-1900 EMCCD (Hamamatsu, Japan) camera using a 5msec 488nm LED illumination pulse. The GFP filter set (DS/FF02-485/20-25, T495lpxr dichroic mirror, and ET525/50 emission filter) was used for Fluo-4 observation.

### INS-1 pancreatic β-cell optical control and calcium imaging

INS-1 cells cultured in RPMI supplemented with 10% FCS, 50μM β-mercaptoethanol, 1mM Sodium pyruvate, 10mM HEPES, 2mM Glutamine and 0.5% penicillin/streptomycin (all Invitrogen) were plated onto 24-well MatTek plates (Ashland, MA, US) at 50,000 cells/well. Cells were incubated at 37°C, 5% CO2 in a humidified chamber for 48 hours prior to viral transduction. Viral transduction for 24 hours at an MOI of 5 preceded imaging by 2 days. Imaging was performed as described above for cardiomyocytes, but with a 10x Olympus UPlanFLN lens (NA 0.3) as cells are more abundant, and lower frequency of activation (0.1–0.3Hz) and lower acquisition frame rate (10Hz) as calcium transient duration is longer. 10 μM Gliclazide was added to Tyrode’s buffer (Sigma-Aldrich- T2145) and imaged from 20 minutes post addition.

### Image processing & statistics

Raw image data was extracted using CellR, and processed in Excel (Microsoft) MATLAB (Mathworks) and OriginLab7.5 (Origin), unfiltered traces are shown. Numerical data is presented as mean +/- standard deviation. Raw movies were exported to Fiji for processing for publication, movies were compressed and time-stamped using VideoMach (Gromada.com). Samples were compared by Students T-test, significance values P<0.05 is shown by *, or P<0.005 by **where applicable.

### Chemicals

Were purchased from Sigma-Aldrich (Dorset, UK) diluted in DMSO, and diluted in culture medium to final concentrations as stated in the text. Cells were observed 30 minutes after drug addition.

## Results and discussion

### Optical control and calcium imaging in primary adult cardiomyocytes

**Fig 1:**
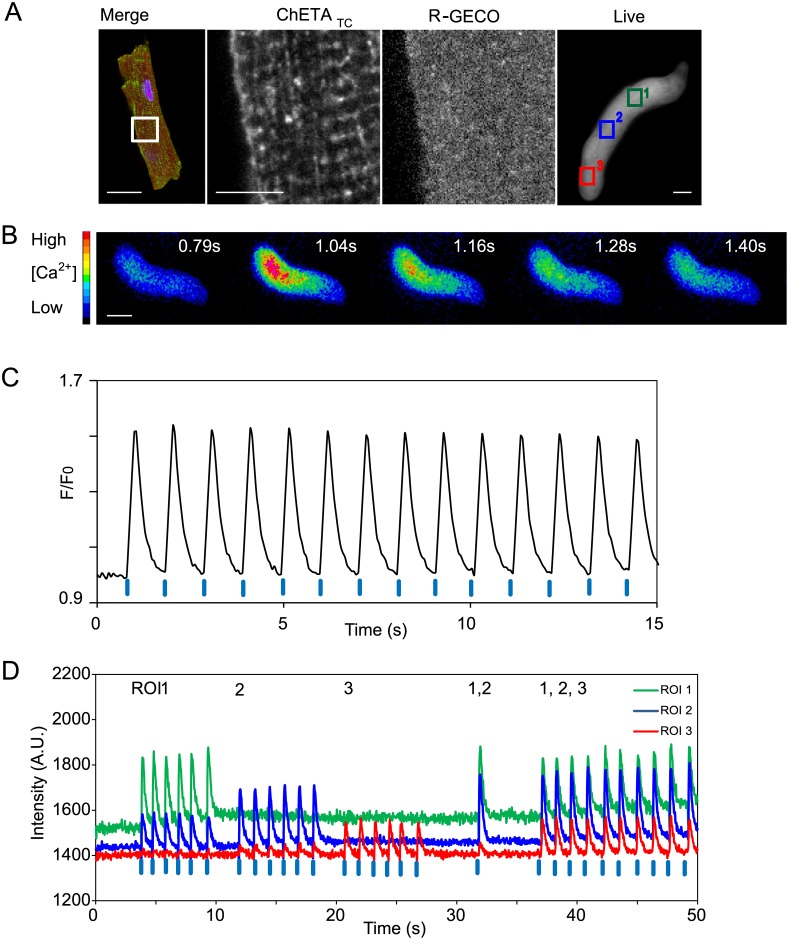
Expression and function of ChETA_TC_ /R-GECO in primary adult cardiomyocytes. (A) Guinea pig ventricular cardiomyocytes were fixed and stained to reveal ChETA_TC_ (green) and R-GECO (red) two days following infection. The magnified region demonstrates regional staining differences compatible with successful 2A peptide cleavage, and membrane localisation of the optical control tool. The panel on the right shows the red fluorescence emission from R-GECO in a living cardiomyocyte; ROI’s show the stimulated regions for Fig 1D. (B) Pseudo coloured image of the single cell calcium transient revealed by R-GECO, stimulation was at t = 1sec (C). Optical stimulation with 405nm light pulses (blue tabs) every second generates synchronised calcium transients in single adult ventricular cardiomyocytes. (D) Subcellular observation and control windows in single cells. ROI’s as shown in Fig 1A were stimulated with 405nm light singly or in combination as documented in the text above the trace. Raw intensity changes for the ROI’s are shown across the experiment. Scale bar = 10μm.

Although not currently possible, changes to hSC-CM differentiation and culture aspire to produce increasingly adult ventricular phenotypes, hence initial method development was undertaken in primary ventricular cardiomyocytes. We felt the brief in vitro survival of adult cells should bias tool selection to the brightest of the indicator/control tool combinations previously tested in neurons. An adenovirus was made containing the optical control tool, ChETA_TC_ [24] engineered for large photocurrents and rapid inactivation, together with the brightest red shifted calcium indicator R-GECO [21,25]. To prevent unbalanced expression of two transgenes in a single cell [26] a rapid self-cleaving 2A peptide [27] was used (S1 Fig). Guinea pig ventricular cardiomyocytes survive 72 hours once isolated allowing 48 hours for reporter production (Fig 1A) after infection. Electrical stimulation confirmed the ability of the calcium indicator to report in this cell-type (S2 Fig). It was also possible to control the cells optically with blue light stimulation and observe the triggered calcium transient by fluorescent red emission (Fig 1B and 1C, S1 Movie). Laser illumination enables control of the size, intensity, and duration of the stimulating light spot. Discrete observation and control windows in single cells can therefore be made (Fig 1D) overcoming imaging artefacts previously tackled by syncytial approaches where optical control, and reporter tools are expressed in different cell populations [14,28].

### Optical control and calcium imaging in stem-cell derived cardiomyocytes

The growth of human genomes represented in iPS repositories, and the ease of hSC-CM differentiation, provides a bridge between computer models and patients [29]. Existing differentiation strategies produce cells with APD and cycle length spanning an order of magnitude [30] particularly at low cell density [31]. This variability may hinder identification of small effect sizes relevant to human health, for example a QT interval of 450msec is normal, whereas 500msec is pathological [32].

**Fig 2:**
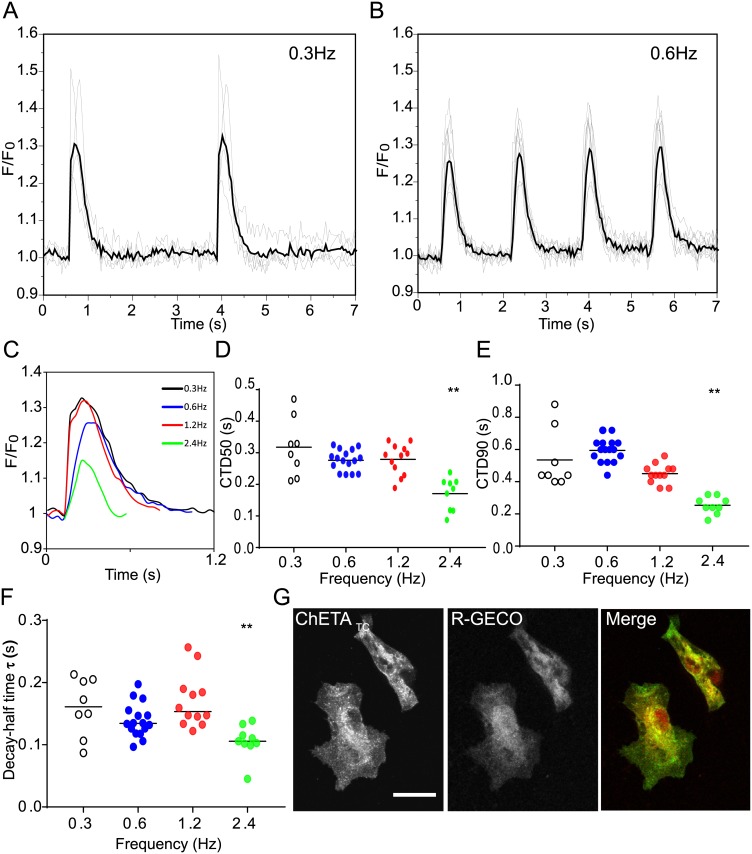
Expression and function of ChETA_TC_ /R-GECO in single stem-cell derived cardiomyocytes. hSC-CM’s were singularised and replated at low density before stimulation at 0.3Hz, 0.6Hz, 1.2Hz, or 2.4Hz with 405nm light, and simultaneous calcium transient visualisation. (A&B) Averaged response is shown by the heavy line, individual cell response by the thin line for 0.3Hz, and 0.6Hz stimulation. (C) Pacing up to 2.4Hz was possible, average results of single calcium transients are shown. (D-F) From the transient it is possible to measure the half maximal width (CTD50), and the 90% transient duration (CTD90), slopes of activation and decay can also be estimated. Individual cell responses are plotted with sample means represented by black bars. (G) Expression analysis of ChETA_TC_ and R-GECO in hSC-CMs does not show the same striated pattern seen in primary cells as these cells lack T-tubule invaginations. Significance values are indicated by * (P = <0.05), and ** (P<0.005) respectively. Scale bar = 10μm.

Although adult cardiomyocytes do not spontaneously depolarise, the hSC-CM’s do (those used here beat at 0.52Hz +/- 0.15Hz). Some cells have lower (<0.2Hz) rates of spontaneous activity. We reasoned this group might represent an electrically consistent population for further testing as spontaneous activity would not break through an applied optical pacing regime. Programmed optical stimulation at lower frequencies (Fig 2A–2C, S2 Movie) gave CTD90 values (Tables 1 and 2, Fig 2E) similar to the reported 1Hz APD90 (0.521 +/- 0.069 sec) for these cells. At higher stimulation frequencies loss of signal to noise, and CTD shortening was apparent (Fig 2C–2F) similar to electrical stimulation using chemical dyes (S3 Fig, and [17]). Immunofluorescence suggests that although membrane localisation of ChETA_TC_ occurs by 48 hours, the development of T-tubule like invaginations in the hSC-CM membrane does not occur (Fig 2g). Importantly calcium sequestration with BAPTA (1mM) blocked significant fluorescent responses at 1.2Hz (S4 Fig) suggesting photoactivation artefact from R-GECO [21] can be avoided. Vehicle addition had no clear effect (S5 Fig). Since lower triggering frequencies minimise potential phototoxicity, and photoactivation artefact, while maximising signal to noise, low frequency stimulation at 0.3Hz and 0.6Hz was used in further work.

**Table 1:** 0.3Hz hSC-CM stimulation results following drug addition summary.

	CTD50 (s)	CTD90 (s)	Calcium change (%)	Rising Slope	Decay time τ (s)
Baseline	0.32+-0.09	0.54+-0.18	38.12+-12.96	1.53+-0.47	0.16+-0.04
Flecainide (0.5 μM)	0.79+-0.07**	1.32+-0.34**	37.28+-17.13	0.72+-0.34*	0.30+-0.01**
Dofetilide (5nM)	0.34+-0.05	0.69+-0.11*	23.83+-8.59	1.03+-0.41*	0.18+-0.01*
Cisapride (30nM)	0.50+-0.14	0.97+-0.31**	32.56+-6.21	2.08+-1.10	0.31+-0.06*
Nifedipine (100nM)	0.23+-0.06**	0.50+-0.11	7.53+-0.44**	-	0.22+-0.04*

Significance values are indicated by

P = <0.05*,

P<0.005**

**Table 2:** 0.6Hz hSC-CM stimulation results following drug addition summary.

	CTD50 (s)	CTD90 (s)	Calcium change (%)	Rising Slope	Decay time τ (s)
Baseline	0.28+-0.03	0.60+-0.07	32.13+-10.1	3.12+-2.12	0.14+-0.02
Flecainide (0.5 μM)	0.81+-0.09**	1.18+-0.20**	38.0+-17.06	0.66+-0.35**	0.43+-0.22**
Dofetilide (5nM)	0.32+-0.02**	0.57+-0.12	28.91+-15.91	2.63+-1.64*	0.20+-0.05**
Diltiazem (1μM)	-	-	3.18+-4.2**	-	-
Verapamil (1μM)	0.26+-0.06	0.42+-0.07**	9.6+-3.1**	1.00+-0.37**	0.20+-0.04**

Significance values are indicated by

P = <0.05*,

P<0.005**

Drug testing can both be done by paired measurements before and after compound addition using the same cell as its own internal control [14, 16]; or as an unpaired assessment compared to a reference population (S6 Fig, and approximately half of the patient disease models summarised in [3]). The first approach might improve sensitivity to small drug effects, whereas the second approach is simpler but vulnerable to intrinsic variability in a sample which may preclude this option. We find that the cell selection strategy combined with optical stimulation limits baseline variability and makes the second approach feasible, although either is possible (S6 Fig).

**Fig 3:**
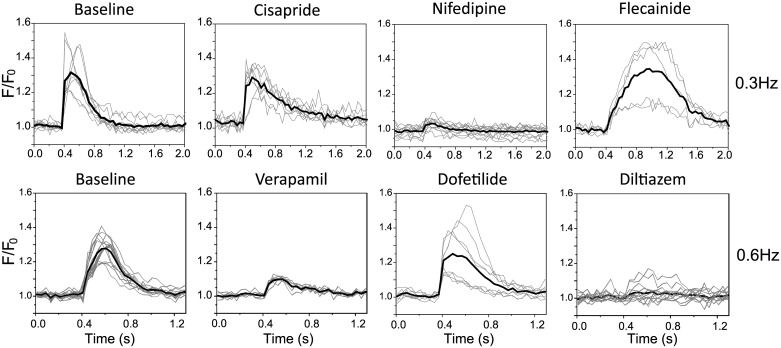
Small molecule ion channel inhibitors applied to hSC-CMs. hSC-CMs were plated out at low density and exposed to clinically relevant doses of known small molecule inhibitors of the sodium channel NaV1.5 (flecainide 0.5μM), the potassium channel KCNH2/hERG (dofetilide, 5nM, and cisapride, 30nM), and the voltage gated calcium channel CACNA1.2 (nifedipine, 100nM; verapamil 1μM, diltiazem 1μM). Traces obtained at 0.3Hz, and 0.6Hz are shown for the annotated small molecule as before with the average response shown by the thick line. Analysis of the data is presented in Tables 1 and 2.

**Fig 4:**
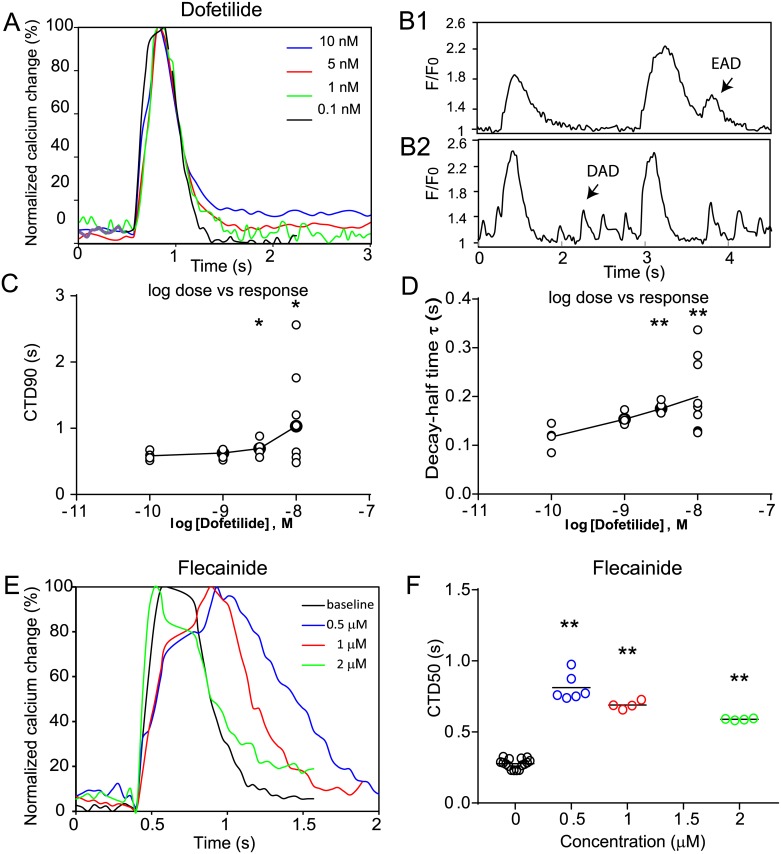
Detailed analysis of dofetilide, and flecainide response in optically controlled hSC-CMs. (A) Averaged response of single hSC-CMs to a Dofetilide dose series. (B) Unfiltered calcium traces from single cells showing dofetilide triggers proarrythmic early after depolarisations (EAD) (B1) and delayed after depolarisations (DAD) (B2). These cells were excluded from the averaged response in(A). (C-D) Log dose response for the CTD90, and decay half time in response to dofetilide is shown. Cells showing EAD, or DAD like perturbations to the calcium transient were excluded. (E) Average dose response to Flecainide is shown, with prolongation of rise time, and calcium transient duration at lower doses which normalises at higher doses. (F) CTD50 changes for the different flecainide doses are shown graphically. Significance values are indicated by * (P = <0.05), and ** (P<0.005) respectively.

Application of flecainide (sodium (INa) channel inhibitor), dofetilide (potassium (hERG) channel inhibitor), cisapride (serotonin receptor inhibitor with off-target hERG block), or the voltage dependent L-type calcium channel inhibitors verapamil (has off target hERG inhibition), nifedipine, and diltiazem were tested at clinically relevant concentrations (Fig 3, Tables 1 and 2). All calcium channel blockers suppress the triggered intensity change of the calcium indicator suggesting this alone may identify such compounds. However loss of indicator brightness represents a weakness in this strategy as although the anticipated CTD shortening with nifedipine was seen, the opposite trend due to off-target hERG block by verapamil was missed even though this was detected in parallel patching studies. Cisapride and dofetilide (Fig 4A, 4C and 4D) both cause dose-dependent prolongation of CTD, with early (Fig 4B1) and after (Fig 4B2) depolarisation transients at higher doses. Flecainide demonstrated a complex response, reducing the activation slope of the calcium transient, and also prolonging it. hERG inhibition by flecainide [33] at this clinical dose is reported, at higher doses this effect is less marked (Fig 4E and 4F) reflecting the integrated sum of other off-target effects.

### Optical control and calcium imaging in pancreatic beta-cells

The need for simultaneous calcium transient phenotyping and control is not a unique cardiomyocyte problem suggesting the method may be useful in other excitable cell types. The beta-cell of the endocrine pancreas uses a glucose triggered voltage change to produce a calcium transient enabling insulin release by exocytosis. Glucose causes ATP levels to rise, increasing the inhibition of an ATP dependent potassium channel K_ATP_ reducing repolarisation, and causing calcium release. Augmentation of insulin release by chemical inhibition of K_ATP_ using the sulphonylurea class of drugs has been a mainstay of Type II Diabetes management for 60 years [34]. Optical control and calcium imaging has been tried in this model [35, 36] but although insulin accumulation could be measured biochemically the underlying calcium transients were either not detected, or drift upward as the excitation spectrum of the calcium indicators (Fluo-4-AM, and Fura2-AM respectively) overlaps with the blue-green activation spectrum of the optical control tool causing unintended ChR2 activation during imaging.

As proof of concept that simultaneous optical control and calcium imaging could be improved the immortalised rat insulinoma beta-cell model (INS-1) was infected and imaged as above in the presence or absence of the sulphonylurea gliclazide at low (3mM) and activating (9mM) glucose concentrations. In the absence of optical control, a chaotic pattern of random calcium activation is apparent (S3 Movie, S7 Fig). At 3mM glucose 45.7 +/- 0.28% of cells have a calcium transient 10% greater than baseline over a 2minute interval, which increases to 71.4 +/- 0.05% at 9mM (p = 0.01); this is accompanied by an increase in transient frequency (1.06 +/- 0.17 to 2.88 +/- 0.3, p<0.001) and increase in peak transient intensity (1.21 +/-0.01 to 1.41 +/- 0.02, p<0.001, n = 110 at 3mM, and 91 at 9mM respectively) but variability in the sample makes extraction of transient parameters challenging.

**Fig 5:**
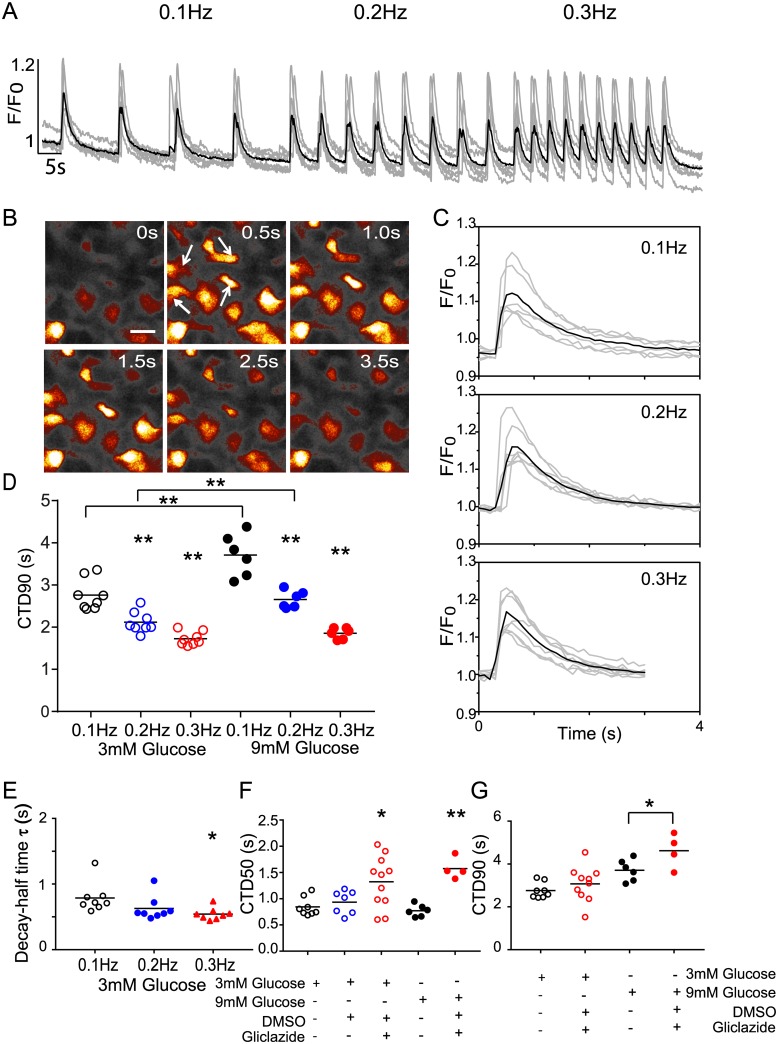
Application of optical control and calcium imaging in pancreatic beta-cells. (A) Intensity traces of the calcium transient visualised by R-GECO in INS-1 beta-pancreatic cells triggered by optical stimulation at frequencies 0.1–0.3Hz, the averaged responses of 10 cells is shown in the black trace. (B) A pseudo-coloured calcium transient time-series taken from movie 3 at 60x. The cells indicated by arrows in the top left of the image were controlled optically, other activity in the field of view is spontaneous. Scale bar = 20μm. (C) Extracted calcium traces at 3mM glucose following 0.1Hz, 0.2Hz, and 0.3Hz activation, the averaged result shown in black. (D) The effect of glucose concentration and stimulation frequency in INS-1 cells determined by CTD90 analysis of calcium intensity traces following optical stimulation. (E) Decay constants of calcium transients in INS-1 cells in 3mM glucose stimulated at 0.1–0.3Hz are shown. (F) CTD50 analysis for INS-1 cells stimulated at 0.1Hz in 3mM and 9mM glucose are shown at baseline, following 1% DMSO or 10μM Gliclazide addition. (G) CTD90 analysis for INS-1 cells stimulated at 0.1Hz in 3mM and 9mM glucose are shown at baseline, or following 10μM Gliclazide addition. Significance values are indicated by * (P = <0.05), and ** (P<0.005) respectively.

By contrast optical control of the calcium transient in beta-cells is possible (S3 Movie) across the range of frequencies observed in spontaneous samples (S7c Fig), at 3mM (Fig 5A–5C) and 9mM glucose (Fig 5D) producing coordinated datasets from which parameters equivalent to the cardiomyocyte can be derived (Fig 5E, Table 3). When exposed to 10μM gliclazide prolongation of CTD50 and CTD90 was observed at 9mM glucose, at 3mM glucose only a change in CTD50 was seen, peak calcium intensity increases in both 3mM and 9mM glucose, as summarised in Table 3 and Fig 5F and 5G.

**Table 3:** Summary of INS1 calcium transient changes due to glucose and gliclazide at 0.1 Hz.

	CTD50 (s)	CTD90 (s)	Calcium change(%)	Decay time τ(s)
3mM glucose baseline	0.85+-0.18	2.76+-0.37	16.00+-0.92	0.79+-0.23
3mM glucose + 10μM gliclazide	1.32+-0.49*	3.07+-0.82	69.60+-17.25**	2.53+-2.39*
9mM glucose baseline	1.06+-0.19	3.71+-0.50	20.00+-7.29	1.11+-028
9mM glucose + 10μM gliclazide	1.57+-0.20*	4.62+-0.79*	36.71+-11.78**	1.65+-0.69

Significance values are indicated by

P = <0.05*,

P<0.005**

## Discussion

The principle limitations of the method in its current form is that although it identifies that cellular behaviour is altered, unlike patching, it is unable to distinguish effects of individual ion currents through which effects might be occurring. As such it is not a direct replacement for patching. Similarly at the moment transgenes are delivered by virus. This means that primary cardiomyocytes infected in vitro are often used towards the end of their natural life as time for gene expression is required with inevitable loss of quality. This can be offset by in vivo infection, and delayed cell isolation, but at the expense of additional procedures such as direct myocardial injection which limits the general utility of the approach. Furthermore, viral infection produces a host cell response that may alter the overall performance of any cell. This should be possible to overcome either via transgenic approaches of gene targeted knock-in or perhaps use of alternative viral strategies engineered to elicit less host cell reaction.

Improving the maturity of stem-cell derived cardiomyocytes will lead to pacing dependence. As these tools are genetically encoded, and equivalents have already been utilised in transgenic models [9,37], genome knock-in lines with constitutive and homogeneous expression may become useful adjuncts to this approach. Indeed, further refinement identifying particular cardiomyocyte subtypes by targeting promoters active in the atrium or ventricle, as demonstrated for lentiviral promoter:transgene combinations in SC-CMs [38] may be possible.

Methodological improvements may arise by combining hyperpolarising and depolarising optical control tools to completely suppress intrinsic activity independently of innovations in differentiation or cell maintenance. Although this would increase the number of cardiomyocytes suitable for study, compatible indicators are currently limiting for this approach [39]. Alternatively, developments allowing multi-parameter (voltage, calcium and contraction) measurements under optical stimulation would enable creation of tools to explore the interaction between these aspects of cardiac physiology in health and disease states and how those changes can be influenced by small molecules.

## Conclusion

This method provides a contactless all genetic single cell assay with temporal stimulation control over the physiological range of cardiomyocytes and pancreatic beta-cells. Anticipated small molecule effects were detected during brief experimental periods on low cell numbers in two model systems. The approach is quick, simple, and can be applied to microscopes with conventional blue/green/red imaging capabilities, using a single virus and isolated cells.

## Supporting information

S1 Appendix Sequence information. Human codon optimised ChETA_TC_ is shown in blue, with a 5xMyc epitope (EQKLISEEDL) tag in grey, the P2A peptide linker (GSGATNFSLLKQAGDVEENPGP) is in bold and underlined. Human codon optimised R-GECO1 is highlighted in Red. Restriction enzyme sites introduced for cloning purposes are in plain text.
(DOCX)

S1 Fig Overview. An adenovirus containing a fusion of the Channel Rhodopsin variant ChETA_TC_ with an N terminal Myc tag linked to the red fluorescent calcium indicator R-GECO via a self-cleaving 2A peptide was made. Following infection gene synthesis, autocatalytic cleavage and chromophore maturation occurs, producing two functional gene products. The minimum collective time for these processes is 24 hours, and we performed experiments at 48hours. ChETA_TC_, a light sensitive voltage channel, allows excitable cells to be depolarised by light in the blue-green range of the visible spectrum. We used 405nm light in these experiments. Calcium transients in excitable cells can be generated by R-GECO which requires green excitation light, producing red emissions. From the evoked calcium transient, it is possible to extract various parameters, as indicated.
(EPS)

S2 Fig Electrical stimulation of primary cardiomyocytes with R-GECO to visualise the calcium transient. A raw unfiltered calcium transient from a single adult guinea pig ventricular cardiomyocyte controlled with 0.5Hz electrode stimulation confirms R-GECO function in primary cardiomyocytes, normalised emission intensity is shown over time.
(EPS)

S3 Fig Electrical stimulation of hSC-CM’s with chemical dye based visualisation of the calcium transient. (A) Averaged single cell calcium transient traces from hSC-CM’s loaded with the Fluo4 calcium dye and stimulated electrically at 0.5Hz, 1Hz, and 2Hz. CTD50 and CTD90 values extracted from the raw traces to generate (S3A) are shown in (S3B) and (S3C). Significance values are indicated by * (P = <0.05), and ** (P<0.005) respectively.
(EPS)

S4 Fig Calcium sequestration with BAPTA prevents visualisation of a dynamic response to optical stimulation. Photoactivation of R-GECO has been documented in response to 488nm light previously. The same phenomenon occurs to a lesser extent with 405nm light. To ensure the triggered responses visualised here are due to calcium release and not imaging artefact, in addition to showing that cells paced electrically report a calcium transient (S2 Fig), and that cells stimulated optically at higher frequency show reduction (imaging artefact would cause an increase) in signal amplitude, CTD50, and CTD90 (Fig 2C–2E) we used the intracellular calcium sequestration agent BAPTA. The two traces during optical stimulation at 1.2Hz obtained from the same cell before (red line) and after (black line) BAPTA addition are shown. If significant photoactivation of R-GECO were occurring this would be expected to be seen as increased signal in the absence of calcium.
(EPS)

S5 Fig Calcium transient duration with vehicle controls evoked by optical stimulation. (A) Averaged calcium transients obtained by optical stimulation at 0.3Hz in the presence of DMSO at 0.1% and 0.001% are shown. (B) CTD50 and (C) Decay half times are shown graphically and numerically in the table.
(EPS)

S6 Fig Paired versus unpaired small molecule assessment. (A) single hSC-CM’s can be phenotyped at baseline, and then restudied following compound addition. Raw data traces of stimulation at 0.3Hz before and after 0.5 μM flecainide addition are shown. (B) & (C) CTD50, and CTD90 data extracted from paired (P) or unpaired (U) experiments using 0.5μM flecainide and 0.3Hz optical stimulation in single hSC-CM’s is shown. Significance values are indicated by * (p<0.05) and ** (p<0.005) respectively. Pairwise comparison of cells at baseline and then after drug addition limits the impact variability between individual cells may produce. However it increases data storage, data processing requirements, slows down throughput and may be more vulnerable to phototoxicity. An alternative strategy compares drug exposed cells to a reference population. This is more useful when cells show a consistent behaviour. We find that the cell selection strategy combined with optical stimulation enables either approach, even at the lowest (0.3Hz) stimulation frequency where variability is greatest. In the paired experiment CTD90 rises from 0.82 +/-0.13s to 1.72 +/- 0.37s (p<0.005), in the unpaired experiment it rises from 0.535+/-0.18s to 1.32 +/- 0.34s (p<0.005).
(EPS)

S7 Fig Spontaneous calcium transients in INS-1 cells. INS-1 cells were infected with ChETA_TC_ and R-GECO and imaged at 2 days in 3mM or 9mM glucose. Cells were imaged at 10Hz for 2 minutes and single cell traces extracted for analysis. A change 10% over baseline was regarded as definite activity. The number of spikes, and the maximum intensity were enumerated. (A) 5 traces for each condition are shown. The most active, and the largest transient traces are shown in addition to 3 other traces with behaviour close to the group average. 53% of the cells at 3mM had no detectable activity, compared to 28% at 9mM. (B) F/Fo intensity, and (C) event rate over 2min are plotted. Significance is indicated by * (p<0.01) and ** (p<0.001).
(EPS)

S1 Movie Calcium transient imaging in an optically stimulated Adult ventricular cardiomyocyte. Isolated Adult ventricular myocytes were infected with the ChETA_TC_ and R-GECO and stimulated with 405nm light pulses at 1Hz with continuous visualisation of the red calcium transient. A Pseudo-coloured movie of a grey-scale image is provided. Time stamp = seconds.
(MP4)

S2 Movie Calcium transient imaging in an optically stimulated hSC-CM. hSC-CMs were infected with the ChETA_TC_ and R-GECO and singularised. Cells were stimulated with 405nm light pulses at 0.3Hz with continuous visualisation of the red calcium transient. A Pseudo-coloured movie of a grey-scale image is provided. Time stamp = seconds.
(MP4)

S3 Movie Calcium transient imaging in INS-1 cells with discrete optical stimulation. INS-1 cells were infected with ChETA_TC_ and R-GECO and visualised at 2 days. Cells in the top left corner marked by an asterix * were stimulated with 405nm light pulses at 0.1Hz, other cells show a random activation. A pseudo-coloured movie of a grey-scale image is provided. Time stamp shows hours:minutes:seconds:milliseconds, the 10Hz acquisition is presented at 50 frames per second.
(MP4)

## Abbreviations

APD: Action Potential Duration
ATP: adenosine tri-phosphate
BAPTA: 1,2-bis(o-aminophenoxy)ethane-N,N,N’,N’-tetraacetic acid
CiPA: Comprehensive In Vitro Proarrhythmia Assay
CTD: Calcium Transient Duration
DMSO: Dimethyl sulfoxide
hERG: human Ether-a-go-go Related Gene—a potassium channel in the heart
hSC-CM: human stem cell derived cardiomyocyte
K_ATP_: a heteroctomeric ATP sensitive potassium channel present in the beta-cell of the pancreas
