# Supplementary material for: Non-invasive phenotyping and drug testing in single cardiomyocytes or beta-cells by calcium imaging and optogenetics
Source: PLoS One. 2017 Apr 5;12(4):e0174181. doi: 10.1371/journal.pone.0174181 (PMC5381843; doi:10.1371/journal.pone.0174181)
Supplement: S1 Appendix — Human codon optimised ChETATC is shown in blue, with a 5xMyc epitope (EQKLISEEDL) tag in grey, the P2A peptide linker (GSGATNFSLLKQAGDVEENPGP) is in bold and underlined. Human codon optimised R-GECO1 is highlighted in Red. Restriction enzyme sites introduced for cloning purposes are in plain text. (DOCX) [file pone.0174181.s001.docx]

**Appendix 1. Sequence Information.**

GGTACCACCATGGATTATGGAGGCGCACTGTCTGCTGTCGGAAGAGAACTGCTGTTTGTGACTAACCCCGTCGTCGTGAATGGGAGCGTGCTGGTCCCCGAGGACCAGTGCTACTGTGCTGGCTGGATCGAATCCAGGGGGACTAACGGAGCCCAGACCGCTTCTAATGTGCTGCAGTGGCTGGCCGCTGGCTTCAGCATCCTGCTGCTGATGTTCTACGCCTATCAGACTTGGAAGTCAACCTGCGGCTGGGAGGAAATCTACGTGTGCGCTATTGAGATGGTGAAGGTCATCCTGGAGTTCTTTTTCGAGTTCAAGAATCCAAGTATGCTGTACCTGGCTACCGGACACCGAGTGCAGTGGCTGCGGTATGCAACATGGCTGCTGACTTGCCCCGTCATCCTGATTCATCTGTCCAACCTGACTGGCCTGAGCAATGACTACTCCCGGAGAACCATGGGGCTGCTGGTGAGTGATATCGGCTGTATTGTCTGGGGGGCAACATCAGCTATGGCAACTGGCTACGTGAAGGTCATCTTTTTCTGCCTGGGACTGTGCTATGGCGCAAACACATTTTTCCACGCAGCCAAGGCCTACATTGAGGGATATCATACAGTGCCTAAAGGCAGGTGCCGCCAGGTGGTCACTGGAATGGCATGGCTGTTTTTCGTCTCTTGGGGGATGTTTCCCATCCTGTTCATTCTGGGACCTGAGGGATTCGGCGTGCTGTCTGTCTACGGGAGTACCGTGGGACACACAATCATTGATCTGATGAGCAAAAATTGTTGGGGCCTGCTGGGGCACTATCTGAGAGTGCTGATCCACGAGCATATCCTGATTCATGGGGACATTCGCAAGACCACAAAACTGAACATCGGCGGGACCGAGATTGAAGTGGAGACACTGGTCGAAGATGAGGCCGAAGCTGGAGCAGTGCCAGCTAGCGGAATGGAGCAGAAGCTGATCAGCGAGGAAGACCTGAACGAGATGGAGCAGAAGCTGATTAGCGAGGAAGATCTGAATGAGATGGAACAGAAGCTGATCTCTGAAGAGGACCTGAATGAAATGGAGCAGAAACTGATTTCTGAGGAAGATCTGAACGAAATGGAACAGAAACTGATTAGCGAAGAAGACCTGAACGAAACTAGC**GGAAGCGGAGCTACTAACTTCAGCCTGCTGAAGCAGGCTGGAGACGTGGAGGAGAACCCTGGACCT**GGTACCACCATGGTAGACTCATCACGTCGTAAGTGGAATAAGGCAGGTCACGCAGTCAGAGCTATAGGTCGGCTGAGCTCACCCGTGGTTTCCGAGCGGATGTACCCCGAGGACGGCGCCCTCAAGAGCGAGATCAAGAAGGGGCTGAGGCTGAAGGACGGCGGCCACTACGCCGCCGAGGTCAAGACCACCTACAAGGCCAAGAAGCCCGTGCAGCTGCCCGGCGCCTACATCGTAGACATCAAGTTGGACATCGTGTCCCACAACGAGGACTACACCATCGTGGAACAGTGCGAACGCGCCGAGGGCCGCCACTCCACCGGCGGCATGGACGAGCTATACAAGGGAGGTACAGGCGGGAGTCTGGTGAGCAAGGGCGAGGAGGATAACATGGCCATCATCAAGGAGTTCATGCGCTTCAAGGTGCACATGGAGGGCTCCGTGAACGGCCACGAGTTCGAGATCGAGGGCGAGGGCGAGGGCCGCCCCTACGAGGCCTTTCAGACCGCTAAGCTGAAGGTGACCAAGGGTGGCCCCCTGCCCTTCGCCTGGGACATCCTGTCCCCTCAGTTCATGTACGGCTCCAAGGCCTACATTAAGCACCCAGCCGACATCCCCGACTACTTCAAGCTGTCCTTCCCCGAGGGCTTCAGGTGGGAGCGCGTGATGAACTTCGAGGACGGCGGCATTATTCACGTTAACCAGGACTCCTCCCTGCAGGACGGCGTATTCATCTACAAGGTGAAGCTGCGCGGCACCAACTTCCCCCCCGACGGCCCCGTAATGCAGAAGAAGACCATGGGCTGGGAGGCTACGCGTGACCAACTGACTGAGGAGCAGATCGCAGAATTTAAAGAGGCTTTCTCCCTATTTGACAAGGACGGGGATGGGACGATAACAACCAAGGAGCTGGGGACGGTGATGCGGTCTCTGGGGCAGAACCCCACAGAAGCAGAGCTGCAGGACATGATCAATGAAGTAGATGCCGACGGTGACGGCACATTCGACTTCCCTGAGTTCCTGACGATGATGGCAAGAAAAATGAATGACACAGACAGTGAAGAGGAAATTAGAGAAGCGTTCCGCGTGTTTGATAAGGACGGCAATGGCTACATCGGCGCAGCAGAGCTTCGCCACGTGATGACAGACCTTGGAGAGAAGTTAACAGATGAGGAGGTTGATGAAATGATCAGGGTAGCAGACATCGATGGGGATGGTCAGGTAAACTACGAAGAGTTTGTCCAAATGATGACAGCGAAGTAGCTCGAG
